# Hormone Fluctuation and Gene Expression During Early Stages of the Hickory Grafting Process

**DOI:** 10.3390/plants14142229

**Published:** 2025-07-18

**Authors:** Qiaoyu Huang, Haixia Liu, Qinyuan Shen, Huwei Yuan, Fuqiang Cui, Daoliang Yan, Wona Ding, Xiaofei Wang, Bingsong Zheng

**Affiliations:** 1 National Key Laboratory for Development and Utilization of Forest Food Resources, Zhejiang A&F University, Hangzhou 311300, China; qiaoyu_huang@163.com (Q.H.); 19157924350@163.com (H.L.); 2024202011016@stu.zafu.edu.cn (Q.S.); hwyuan@zafu.edu.cn (H.Y.); fuqiang.cui@zafu.edu.cn (F.C.); liangsie@zafu.edu.cn (D.Y.); 2 Provincial Key Laboratory for Non-Wood Forest and Quality Control and Utilization of Its Products, Zhejiang A&F University, Hangzhou 311300, China; 3 College of Science and Technology, Ningbo University, Ningbo 315300, China; dwn@zju.edu.cn

**Keywords:** hormonal crosstalk, callus proliferation, vascular differentiation, graft union formation

## Abstract

Grafting involves complex hormonal interactions at graft interfaces that are not yet fully understood. In this study, we analyzed hormone fluctuations and gene expression during callus proliferation and vascular tissue differentiation in hickory (*Carya cathayensis* Sarg.) grafts. Cytokinin and ethylene precursor ACC levels steadily increased after grafting. The biosynthetic genes for these hormones (*IPT3*, *ACS1*, *ACO1*, and *ACO5*) exhibited heightened expression. Genes related to cytokinin signaling (*RR3*, *ARR4*, and *ZFP5*) and ethylene signaling (*MKK9*, *ESE1*, and *ESE3*) were similarly upregulated. Conversely, genes associated with jasmonic acid, abscisic acid, and strigolactone pathways were downregulated, including synthesis genes (*AOC4* and *AOS*) and those involved in signal transduction (*NAC3*, *WRKY51*, and *SMAX1*). Correspondingly, JA-Ile and 5-deoxystrigol levels significantly decreased. Indole-3-acetic acid (IAA) levels also dropped during the early stages of graft union formation. These results suggest that low auxin concentrations may be essential in the initial stages after grafting to encourage callus proliferation, followed by an increase at later stages to facilitate vascular bundle differentiation. These findings imply that maintaining a balance between low auxin levels and elevated cytokinin and ethylene levels may be critical to support cell division and callus formation during the initial proliferation phase. Later, during the vascular differentiation phase, a gradual rise in auxin levels, accompanied by elevated ethylene, may facilitate the differentiation of vascular bundles in hickory grafts.

## 1. Introduction

Grafting represents a fundamental horticultural technique wherein a branch or bud is joined to the stem or root of another plant to generate a new, grafted individual [1]. This method is extensively utilized in fruit and vegetable seedling production and serves a vital role in agricultural research [2,3]. Successful grafting requires the formation of callus tissue connecting the initially separate scion and rootstock, with callus formation indicating compatibility between graft partners [4]. The reconstruction of vascular tissue at the graft site is essential for graft viability [5], and grafting triggers physiological responses related to wounding hormones and signaling pathways critical for vascular tissue regeneration and attachment [6,7]. This response enhances the activity of genes related to auxin, a key hormone in vascular development [8]. Auxin distribution is regulated by polar auxin transport controlled by PIN and ABCB proteins. Activation of PIN1 and ABCB1 genes is vital for graft development, as these genes facilitate auxin efflux and transport [9].

The results of a number of studies demonstrate the significance of additional phytohormones, including cytokinins (CK), ethylene (ETH), and abscisic acid (ABA), in the grafting process [10,11,12,13]. These hormones interact with auxins to regulate plant growth and development. Research has highlighted that CK influence auxin transport during vascularization by regulating PIN protein distribution within nascent vascular tissues in *Arabidopsis* [14]. In pecan (*Carya illinoinensis* (Wangenh.) K. Koch) grafting, a B-type *Arabidopsis* response regulator (ARR) functions as a primary regulator of callus proliferation at the graft junction, mediated by CK [15]. Incision of the grafted surface initiates a wounding stress response that is strongly regulated by the jasmonic acid (JA) signaling pathway. A substantial increase in JA levels occurs within 30 s of wounding in *Arabidopsis*, with changes in JA-Ile levels detected within 5 min of wounding [16]. JA additionally serves as a crucial long-distance signal in the wound signaling pathway and plays an essential role in promoting union at the graft interface [17].

Transcriptomic analyses have revealed that ETH biosynthesis genes are activated in grafts of *Arabidopsis* hypocotyls [18] and tobacco [19]. Application of the ETH precursor 1-aminocyclopropane-1-carboxylate (ACC) enhanced scion growth in tobacco grafts; in comparison, the ETH inhibitor aminoethoxyvinylglycine (AVG) impeded graft union and diminished scion growth [19], demonstrating the positive role of ETH in grafting. Synergistic effects between hormone classes have been observed, with IAA, GA (gibberellin), and CK promoting cell differentiation, vascular bundle formation, and xylem/phloem reconnection [20]. Similarly, strigolactones (SL) operate in conjunction with auxin and CK to regulate plant branching [21]. However, comprehensive quantification of hormones and related compounds in their biosynthetic pathways at the rootstock–scion graft junction remains an emerging research area.

Hickory is a valuable nut tree native to China. The hickory industry is constrained by numerous challenges, including slow plant growth, excessive tree height, restricted habitat, and seed cultivation complications. Grafting serves as a method to enhance the adaptability and production of hickory [22]. The authors of previous studies have explored the role of auxin in hickory grafting, examining auxin response factors (*ARFs*) [23], *AUX*/*IAA* [24], *CcGH3* [25], *TIR1*/*AFB* [26], *CcPIN* [27], and *CcABCB* [28]. However, a comprehensive understanding of hormonal changes and molecular mechanisms supporting graft survival remains limited. Based on the results of our previous study [22], we analyzed hormone levels in hickory grafts at various time points using LC–MS/MS to elucidate their role in graft establishment. In addition, we utilized transcriptomic data to examine changes in hormone-related gene expression during early graft union formation.

## 2. Results

### 2.1. Variations in Endogenous Hormone Levels During Different Phases of Hickory Grafting

**Figure 1:**
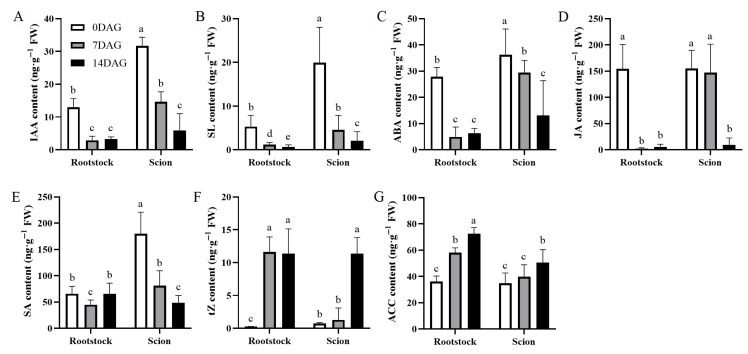
Temporal dynamics of phytohormone accumulation in the rootstocks and scions during graft healing. Seven phytohormones (**A**–**G**) measured in the rootstocks and scions at three key stages of graft development: 0 DAG (white bars), 7 DAG (gray bars), and 14 DAG (black bars). Each panel represents a specific hormone: (**A**) IAA, (**B**) tZ, (**C**) ACC, (**D**) JA, (**E**) SA, (**F**) SL, and (**G**) ABA. Values are expressed as ng·g^−1^ fresh weight (FW). The data is presented as means ± SE (the standard error) (*n* = 3 biological replicates). Different lowercase letters above bars indicate statistically significant differences between time points within each tissue type (one-way analysis of variance ANOVA, Tukey’s test, *p* < 0.05).

In previous studies on hickory grafting, researchers have identified 7 and 14 days after grafting (DAG) as crucial stages for morphological changes, including necrotic layer formation, callus growth, and new vascular tissue development [22,29]. In this study, we quantified phytohormone concentrations in hickory rootstocks and scions from 0 to 14 DAG. During the initial phase following hickory grafting, 7 and 14 DAG, endogenous auxin levels exhibited a marked decrease (Figure 1A). Similarly, SL and ABA concentrations significantly decreased during this period (Figure 1B,C). JA content changes demonstrated tissue specificity; in the rootstock, there was a rapid and substantial decrease immediately after grafting; in the scion, in comparison, a notable reduction was observed only at 14 days post-grafting (Figure 1D). Salicylic acid (SA) level fluctuations differed markedly between the rootstocks and scions. Post-grafting, rootstock SA content remained relatively stable; in the scion, in comparison, a significant decrease was recorded (Figure 1E). In contrast, trans-Zeatin riboside (tZ) levels increased significantly in the rootstock after grafting, with the scion exhibiting a substantial increase at 14 DAG (Figure 1F). In addition, the pattern of change in 1-aminocyclopropane-1-carboxylic acid (ACC) paralleled that of CK. Post-grafting, rootstock ACC content increased substantially, with that of the scion showing significant elevation 14 days thereafter (Figure 1G).

### 2.2. Modifications of Auxin Metabolism and Signaling Pathways

**Figure 2:**
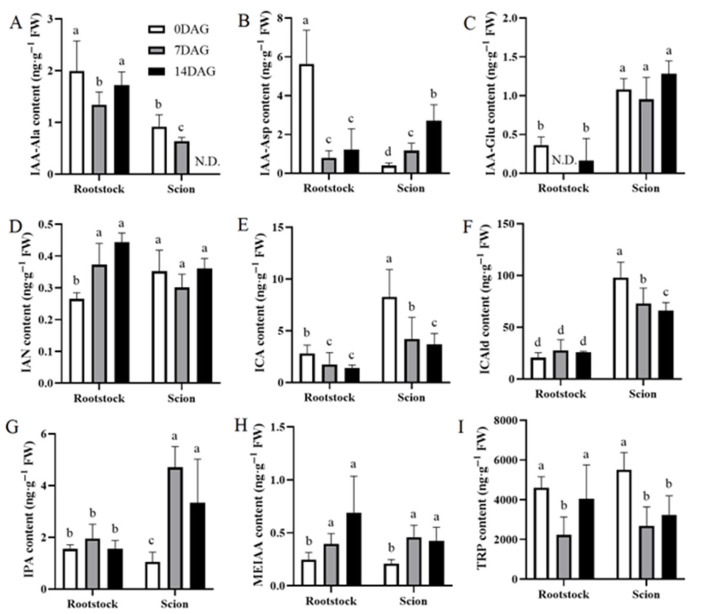
Temporal dynamics of auxin metabolites accumulation in the rootstocks and scions during graft healing. Nine auxin metabolites (**A**–**I**) measured in the rootstocks and scions at three key stages of graft development: 0 DAG (white bars), 7 DAG (gray bars), and 14 DAG (black bars). Each panel represents an auxin metabolite: (**A**) IAA-Ala, (**B**) IAA-Asp, (**C**) IAA-Glu, (**D**) IAN, (**E**) ICA, (**F**) ICAld, (**G**) IPA, (**H**) MEIAA, and (**I**) TRP. Values are expressed as ng·g^−1^ FW. The data is presented as means ± SE (the standard error). Different lowercase letters above bars indicate statistically significant differences between time points within each tissue type (one-way ANOVA, Tukey’s test, *p* < 0.05).

Auxin plays a crucial role in vascular tissue development, which is essential for successful graft union formation [13]. We analyzed auxin and its associated metabolites concentrations in rootstocks and scions at 0, 7, and 14 DAG to understand their contribution to grafting and vascular bundles integration in hickory (Figure 2A–G). Among IAA amino acid conjugates, IAA-alanine (IAA-Ala) demonstrated initial accumulation (0 DAG) followed by a gradual decline in the scion, with IAA-aspartate (IAA-Asp) exhibiting an increase (Figure 2A,B). IAA-Glu levels remained stable in the scion and exceeded rootstock levels (Figure 2C). Indole-3-acetonitrile (IAN) concentrations remained relatively constant in both the rootstocks and scions (Figure 2D). Indole-3-carboxylic acid (ICA) and indole-3-carbaldehyde (ICAld), byproducts of tryptophan (TRP) metabolism, exhibited continuous downregulation in scion tissues with temporal coordination to the declining TRP pool (Figure 2E,F,I). Indole-3-pyruvic acid (IPA) content exhibited significant increases at both 7 DAG and 14 DAG compared to 0 DAG in the scions (Figure 2G). Methoxy-indole-3-acetic acid (MEIAA), a methionine derivative of IAA, demonstrated a negative correlation with TRP, suggesting potential involvement in IAA signal termination or transport regulation (Figure 2H). TRP, an IAA synthesis precursor, showed high initial expression in the rootstock followed by gradual decline (Figure 2I).

**Figure 3:**
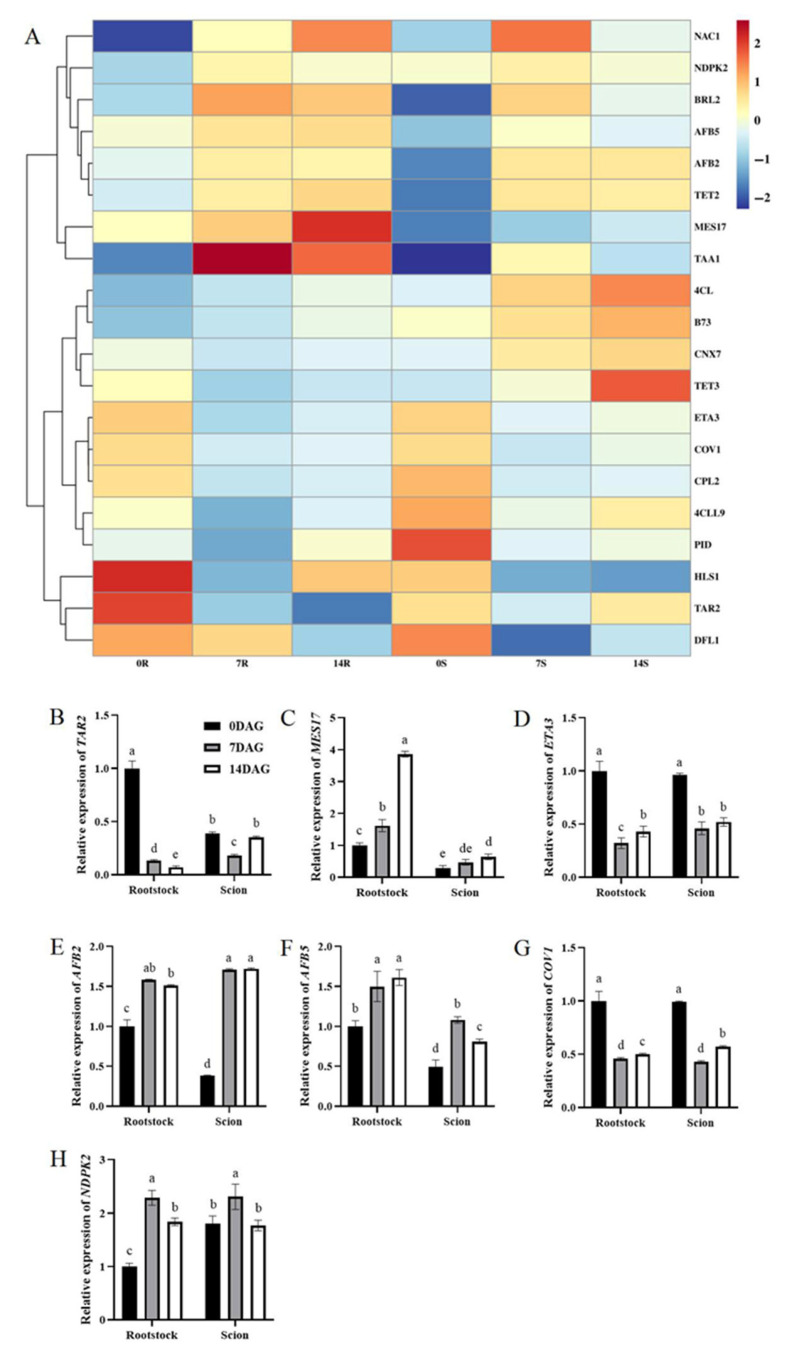
Differential expression genes related to auxin biosynthesis and signal transduction during graft union development. (**A**) Expression heatmap of differential expression genes related to auxin biosynthesis and signal transduction during graft union development. Log2 and row scale normalization were applied to the RNA-seq-based FPKM values, and heatmap visualization was performed using TBtools (v2.310). The numbers 0, 7, and 14 represent different time points (0, 7, and 14 DAG). The letters R and S represent the rootstocks and scions. Color intensity reflects normalized expression values, with a red-to-blue gradient indicating high-to-low transcriptional activity. Expression of *TAR2* (**B**), *MES17* (**C**), *ETA3* (**D**), *AFB2* (**E**), *AFB5* (**F**), *COV1* (**G**), and *NDPK2* (**H**) in hickory rootstocks and scions. The expression level of hickory *ACTIN* gene was used as the internal control to standardize the RNA samples for each reaction. The data is presented as means ± SE. Different lowercase letters indicate significant differences at *p* < 0.05.

Analysis of gene expression patterns provided further insights into auxin dynamics during graft formation (Figure 3A). Consistent with decreased IAA levels, the auxin biosynthesis gene tryptophan aminotransferase related 2 (*TAR2*) exhibited downregulation in rootstock from 0 to 14 DAG. In contrast, methylesterase 17 (*MES17*), which encodes a protein that converts inactive MeIAA to active IAA, exhibited upregulation during graft formation in both scions and rootstocks (Figure 2C). The genes encoding F-box auxin receptors *AFB5* and *AFB2*, which are essential regulators of auxin perception, showed upregulation in rootstocks and scions throughout graft union formation. In addition, nucleoside diphosphate kinase 2 (*NDPK2*), encoding a protein that modulates auxin transport, exhibited upregulation during graft formation, notably in rootstocks. Activation of auxin signaling pathways involves complex regulatory cascades. Accordingly, the expression levels of auxin-activated signaling pathway genes *COV1* and *ETA3* exhibited significant downregulation at 7 and 14 DAG. qRT-PCR validation confirmed these expression patterns across the examined genes, revealing that *TAR2*, *ETA3*, and *COV1* were upregulated after grafting in both rootstocks and scions at 7 and 14 DAG; in comparison, *MES17*, *AFB2*, *AFB5*, and *NDPK2* exhibited corresponding downregulation (Figure 3B–H). These expression trends closely align with transcriptomic data.

### 2.3. Modifications of CK Metabolism and Signaling

**Figure 4:**
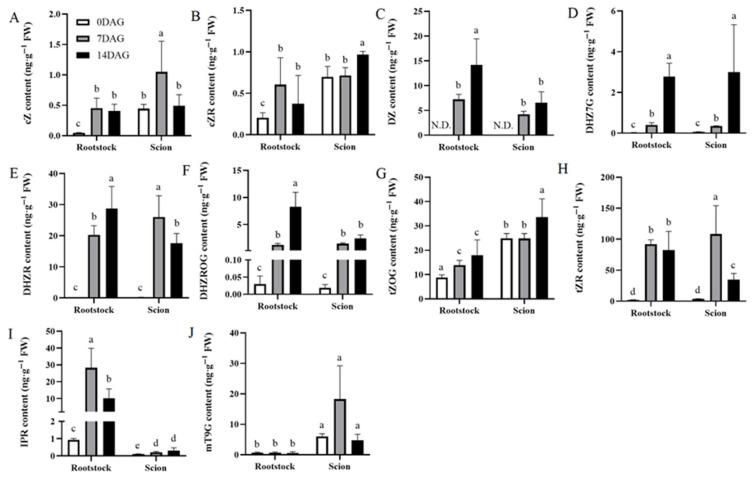
Temporal dynamics of CK metabolite accumulation in rootstocks and scions during graft healing. Ten CK metabolites (**A**–**J**) measured in rootstocks and scions at three key stages of graft development: 0 DAG (white bars), 7 DAG (gray bars), and 14 DAG (black bars). Each panel represents a CK metabolite: (**A**) cZ, (**B**) cZR, (**C**) DZ, (**D**) DHZ7G, (**E**) DHZR, (**F**) DHZROG, (**G**) tZOG, (**H**) tZR, (**I**) IPR, and (**J**) mT9G. Values are expressed as ng·g^−1^ FW. The data is presented as means ± SE. Different lowercase letters above bars indicate statistically significant differences between time points within each tissue type (one-way ANOVA, Tukey’s test, *p* < 0.05).

CK are plant hormones derived from adenine. They are involved in various aspects of plant development, including cell division, lateral root formation, and meristem maintenance. The wound healing process during grafting is similar to callus formation in wounded plants. Our analysis results highlight an increased cytokinin response during wound healing in hickory grafting (Figure 1F). Quantitative analysis demonstrated coordinated induction of multiple CK metabolites including: cis-zeatin (cZ), cis-zeatin riboside (cZR), cZ-riboside dihydrozeatin (DZ), dihydrozeatin ribonucleoside (DZHR), dihydrozeatin-7-glucoside (DHZ7G), dihydrozeatin-O-glucoside riboside (DHZROG), N6-isopentenyladenosine (IPR), trans-zeatin-O-glucoside (tZOG), and trans-zeatin riboside (tZR), all exhibiting significant accumulation compared to 0 DAG (Figure 4A–I). DZ exhibited the most pronounced change, with it being undetectable at 0 DAG but showing progressive accumulation at 7 and 14 DAG in both rootstocks and scions (Figure 4C). In contrast, meta-Topolin-9-glucoside (mT9G) levels remained constant throughout the experimental period in both the scions and rootstocks (Figure 4J).

**Figure 5:**
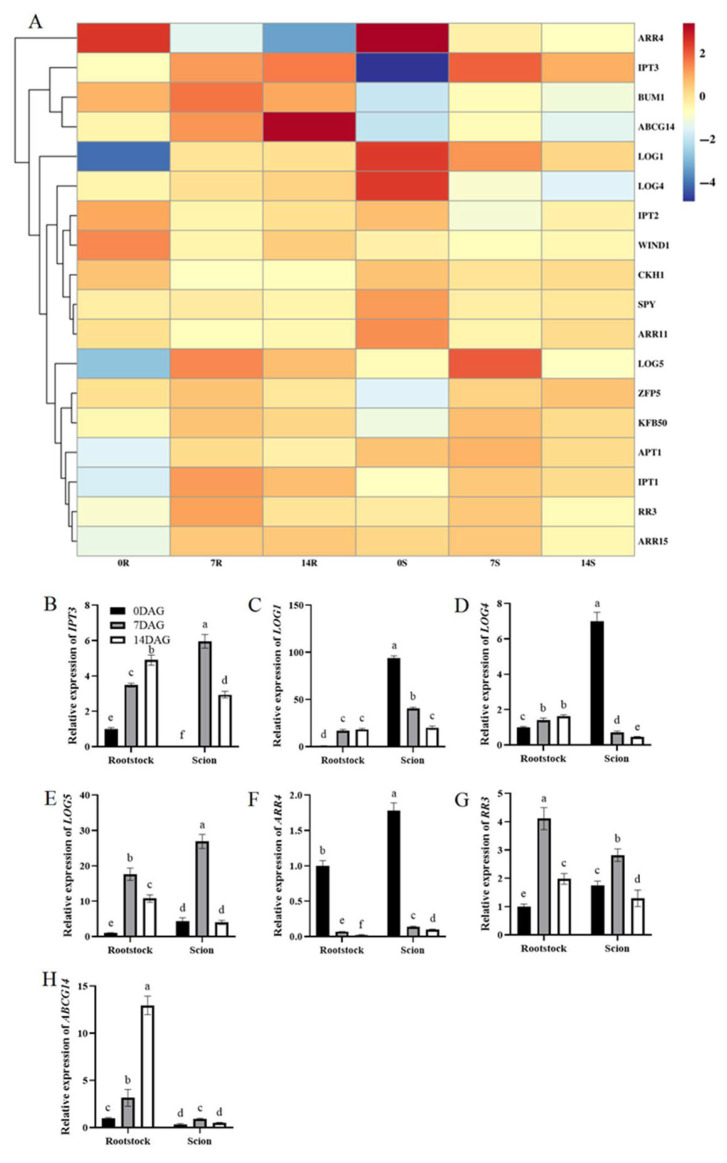
Differential expression genes related to CK biosynthesis and signal transduction during graft union development. (**A**) Expression heatmap of differential expression genes related to CK biosynthesis and signal transduction during graft union development. Log2 and row scale normalization were applied to the RNA-seq-based FPKM values, and heatmap visualization was performed using TBtools (v2.310). The numbers 0, 7, and 14 represent different time points (0, 7, and 14 DAG). The letters R and S represent rootstocks and scions. Color intensity reflects normalized expression values, with a red-to-blue gradient indicating high-to-low transcriptional activity. Expression of *IPT3* (**B**), *LOG1* (**C**), *LOG4* (**D**), *LOG5* (**E**), *ARR4* (**F**), *RR3* (**G**), and *ABCG14* (**H**) in hickory rootstocks and scions. The expression level of hickory *ACTIN* was used as the internal control to standardize the RNA samples for each reaction. The data is presented as means ± SE. Different lowercase letters indicate significant differences at *p* < 0.05.

Our RNA-seq analysis results revealed significant upregulation of *IPT3*, which encodes key enzymes catalyzing the rate-limiting step in CK biosynthesis. Notably, *IPT3* expression exhibited upregulation at 7 and 14 DAG in both scions and rootstocks (Figure 5A). CK transport exhibited enhancement through elevated *ABCG14* expression in rootstocks after grafting. Furthermore, components of CK signaling pathways demonstrated coordinated induction. Specifically, type-A response regulators *RR3* and *ARR4* exhibited upregulation in both scions and rootstocks; in comparison, zinc finger protein *ZFP5* exhibited similar expression patterns. Multiple *LOG* genes (*LOG1*, *LOG4*, and *LOG5*) encoding enzymes essential for CK activation through riboside hydrolysis displayed dynamic expression changes during grafting in rootstocks and scions [30]. qRT-PCR analysis validated these expression profiles, confirming that *IPT3*, *LOG5*, *RR3*, and *ABCG14* demonstrated sustained upregulation after grafting in both rootstocks and scions at 7 and 14 DAG; in comparison, *RR4* exhibited reciprocal downregulation. Notably, *LOG1* and *LOG4* displayed upregulation in rootstocks contrasting with downregulation observed in scions (Figure 5B–H). These expression patterns demonstrated concordance with transcriptomic data.

### 2.4. Dynamics of JA Metabolism and Signaling

**Figure 6:**
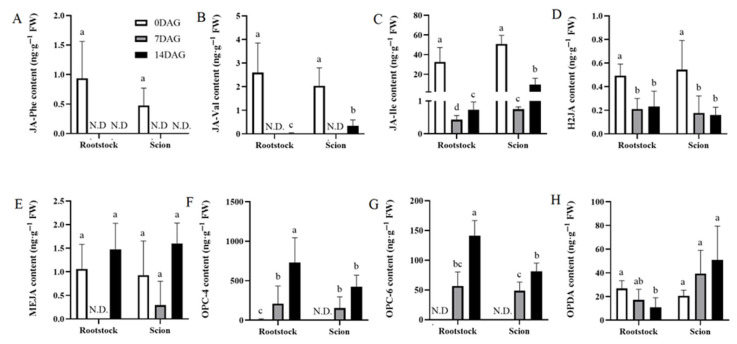
Temporal dynamics of JA metabolites accumulation in rootstocks and scions during graft healing. Eight JA metabolites (**A**–**H**) measured in rootstocks and scions at three key stages of graft development: 0 DAG (white bars), 7 DAG (gray bars), and 14 DAG (black bars). Each panel represents a CK metabolite: (**A**) JA-Phe, (**B**) JA-Val, (**C**) JA-Ile, (**D**) H2JA, (**E**) MEJA, (**F**) OPC-4, (**G**) OPC-6, and (**H**) OPDA. Values are expressed as ng·g^−1^ FW. The data is presented as means ± SE. Different lowercase letters above bars indicate statistically significant differences between time points within each tissue type (one-way ANOVA, Tukey’s test, *p* < 0.05).

JA serves as a crucial long-distance signaling mediator in response to both biotic and abiotic stresses [31], in addition to wound signaling pathways [16]. Quantitative analysis of JA metabolites revealed a systemic reduction in JA levels across both rootstocks and scions post-grafting. Conjugated forms such as JA-Phe, JA-Val, and JA-Ile exhibited coordinated downregulation, with JA-Val becoming undetectable after grafting (Figure 6A–C). Notably, levels of H2JA, a bioactive conjugate involved in JA homeostasis, also decreased after grafting (Figure 6D). Oxophytodienoic acid (OPDA) exhibited a slight reduction in rootstocks but remained stable in scions, corresponding to the unchanged levels of methyl jasmonate (MEJA) (Figure 6E,F). Of note, oxidative JA derivatives OPC-4 and OPC-6 demonstrated time-dependent accumulation, peaking at later grafting stages (Figure 6G,H).

**Figure 7:**
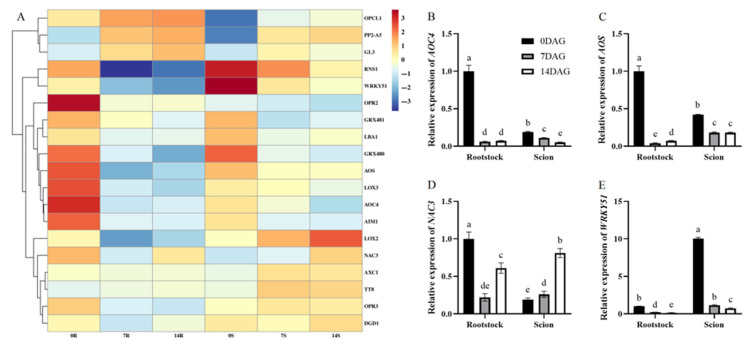
Differential expression genes related to JA biosynthesis and signal transduction during graft union development. (**A**) Expression heatmap of differential expression genes related to JA biosynthesis and signal transduction during graft union development. Log2 and row scale normalization were applied to the RNA-seq-based FPKM values, and heatmap visualization was performed using TBtools (v2.310). The numbers 0, 7, and 14 represent different time points (0, 7, and 14 DAG). The letters R and S represent rootstocks and scions. Color intensity reflects normalized expression values, with a red-to-blue gradient indicating high-to-low transcriptional activity. Expression of *AOC4* (**B**), *AOS* (**C**), *NAC3* (**D**), and *WRKY51* (**E**) in hickory rootstocks and scions. The expression level of hickory *ACTIN* was used as the internal control to standardize the RNA samples for each reaction. The data is presented as means ± SE. Different lowercase letters indicate significant differences at *p* < 0.05.

Consistent with the measured JA levels, our transcriptome analysis results revealed significant downregulation of the JA biosynthesis gene allene oxide cyclase 4 (*AOC4*), particularly in rootstocks during the callus formation stage (Figure 7A). Genes encoding allene oxide synthase (*AOS*), which catalyzes the initial step in JA biosynthesis, exhibited downregulation expression profiles. Several transcription factors associated with JA signaling pathways also downregulated downregulation. Notably, *NAC3* (a known JA-responsive gene [32]) exhibited marked downregulation in rootstocks during 0–7 DAG. Similarly, *WRKY51*, which mediates JA-induced defense responses [33], exhibited significant repression in both tissues throughout the 14-DAG (Figure 7A). This coordinated downregulation of JA biosynthesis and signaling components likely reflects an adaptive mechanism that attenuates defense responses to facilitate successful graft union formation and tissue repair during early developmental stages. Our qRT-PCR analysis results confirmed these expression trends, confirming that *AOC4*, *AOS*, and *WRKY51* showed sustained downregulation after grafting at both 7 DAG and 14 DAG; in comparison, *NAC3* exhibited downregulation in rootstocks, contrasting with upregulation in scions (Figure 7B–E). These expression patterns demonstrate agreement with transcriptomic data.

### 2.5. Dynamics of ETH Metabolism and Signaling

Consistent with its established role in plant wound responses and cell expansion, exogenous ETH application enhances callus formation and cell proliferation [34]. Analysis of ACC levels, the immediate ETH precursor, revealed progressive accumulation in rootstock tissues across the grafting timeline. The ACC concentration reached 75 ng·g^−1^ at 14 DAG, representing a two-fold increase relative to day 0 levels (Figure 1G). Scion tissues exhibited a parallel temporal increase in ETH content.

**Figure 8:**
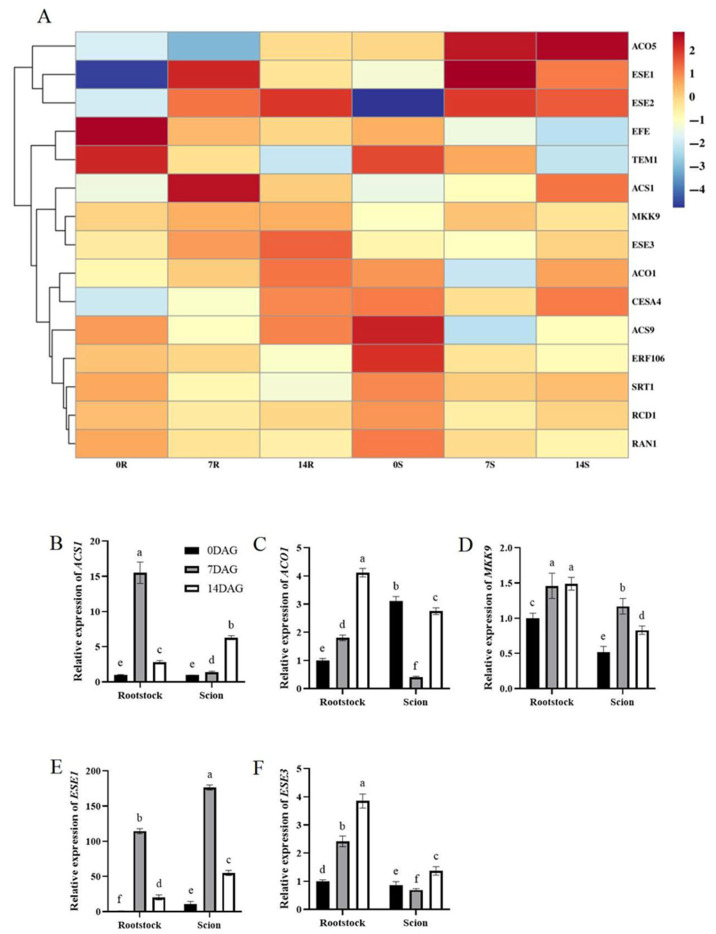
Differential expression genes related to ETH biosynthesis and signaling transduction during graft union development. (**A**) Expression heatmap of differential expression genes related to JA biosynthesis and signaling transduction during graft union development. Log2 and row scale normalization were applied to the RNA-seq-based FPKM values, and heatmap visualization was performed using TBtools (v2.310). The numbers 0, 7, and 14 represent different time points (0, 7, and 14 DAG). The letters R and S represent rootstocks and scions. Color intensity reflects normalized expression values, with a red-to-blue gradient indicating high-to-low transcriptional activity. Expression of *ACS1* (**B**), *ACO1* (**C**), *MKK9* (**D**), *ESE1* (**E**), and *ESE3* (**F**) in hickory rootstocks and scions. The expression level of hickory *ACTIN* was used as the internal control to standardize the RNA samples for each reaction. The data is presented as means ± SE. Different lowercase letters indicate significant differences at *p* < 0.05.

These biochemical changes were correlated with the transcriptomic upregulation of the ETH biosynthesis gene 1-amino-cyclopropane-1-carboxylic acid synthase 1 (*ACS1*) in both graft tissues. ACS catalyzes the rate-limiting step in ETH synthesis by producing ACC, which is subsequently converted to ETH by ACC oxidase (ACO). Notably, *ACO1* expression increased in rootstocks during graft formation, with *ACO5* exhibiting upregulation in scions (Figure 8A). Key regulators of ETH signaling also demonstrated significant induction. The *MAPKK* family member *MKK9*, which modulates ETH biosynthesis and calcineurin signaling, demonstrated substantial upregulation, as did the ETH-responsive *ERF*/*AP2* transcription factors *ESE1* and *ESE3* (Figure 8A). *MKK9* activation triggers the expression of multiple stress-related genes including *ACS2*, *ACS6*, and cytochrome P450 enzymes involved in phytoalexin biosynthesis [35]. Our qRT-PCR analysis results validated these expression dynamics by confirming that *ACS1*, *MKK9*, and *ESE1* exhibited sustained upregulation in both rootstocks and scions at 7 and 14 DAG (Figure 8B,D,E). In contrast, while *ACO1*, and *ESE3* showed rootstock predominant upregulation (Figure 8C,F). These expression patterns correspond closely with the transcriptomic data.

### 2.6. Modifications of SL Metabolism and Signaling

**Figure 9:**
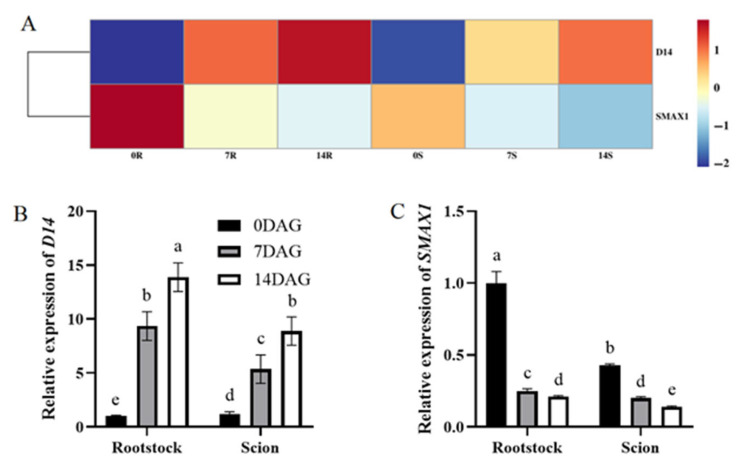
Differential expression genes related to SL biosynthesis and signal transduction during graft union development. (**A**) Expression heatmap of differential expression genes related to SL biosynthesis and signal transduction during graft union development. Log2 and row scale normalization were applied to the RNA-seq-based FPKM values, and the heatmap visualization was performed using TBtools (v2.310). The numbers 0, 7 and 14 represent different time points (0, 7, and 14 DAG). The letter R and S represent rootstock and scion. Color intensity reflects normalized expression values, with a red-to-blue gradient indicating high-to-low transcriptional activity. Expression of *D14* (**B**) and *SMAX1* (**C**) in hickory rootstocks and scions. The expression level of hickory *ACTIN* was used as the internal control to standardize the RNA samples for each reaction. The data is presented as means ± SE. Different lowercase letters indicate significant differences at *p* < 0.05.

SL have been shown to modulate cambial activity via an auxin-dependent mechanism, suggesting their potential involvement in the grafting process [36]. A pronounced decrease in SL concentration was observed after grafting, particularly at 7 DAG, when SL levels decreased by approximately 4.5- and 8.4-fold in the rootstock and scion, respectively (Figure 1B). Transcriptomic profiling revealed coordinated changes in SL pathway genes. *DWARF14* (*D14*), which encodes an α/β hydrolase essential for SL perception, exhibited significant upregulation at both 7 and 14 DAG. Concurrently, the SL-responsive suppressor gene *SMAX1* displayed marked downregulation during the same period (Figure 9A). The expression patterns of *D14* and *SMAX1* were further validated by qRT-PCR analysis (Figure 9B,C). These molecular changes align with physiological observations. Reduced SL levels appear to alleviate signaling repression, thereby promoting cell proliferation and callus formation at the graft interface [37]. This regulatory mechanism may facilitate vascular reconnection and tissue integration during early graft union establishment.

## 3. Discussion

### 3.1. Auxin-Induced Vascular Tissue Formation and the Graft Union Process

Auxins play essential roles in vascular tissue formation and wound union processes, particularly in the differentiation of cambium into secondary phloem and secondary xylem, which requires high auxin concentrations [13,38]. During the initial stage of hickory grafting, the concentration of IAA, the principal form of auxin, decreased by 78% in rootstocks and 24% in scions, accompanied by significant reductions in the auxin precursor Trp. This decline in IAA levels aligns with transcriptomic evidence showing decreased expression of auxin synthesis pathway genes, demonstrating that the graft site had not yet progressed to vascular bundle differentiation. This observation aligns with anatomical studies that have identified callus formation as a preliminary phase preceding vascular reconnection [22]. The observed downregulation of *TAR2* (Figure 3B) in rootstocks further confirms reduced auxin biosynthesis during early grafting. However, the concurrent upregulation of *MES17* (Figure 3C)—which converts inactive MeIAA to active IAA—suggests a compensatory mechanism. This two-part regulatory strategy involves *TAR2* suppression to lower new auxin production and *MES17* activation to recycle stored auxin, ensuring sufficient auxin for early wound responses while maintaining homeostasis.

The wounding phase, characterized by isolation layer formation at the rootstock-scion junction, precedes callus proliferation and vascular differentiation. Similar reductions in IAA at the graft unions have been documented in pecan [15] and litchi (*Litchi chinensis* Sonn.) [39], with low IAA concentrations predominantly inducing phloem differentiation, whereas high concentrations promote both phloem and xylem [9]. Notably, IAA levels recover, coinciding with the peak of xylem differentiation [9]. Early auxin responsive genes, including *AUX*/*IAA*, *GH3*, and *PIN* families, significantly impact signaling pathways [24,25,27]. The *GH3* gene family regulates auxin homeostasis, potentially through suppression during the early grafting stages, which may result from inhibited amino acid synthesis (e.g., IAA-Asp) and subsequent free IAA release [25]. P The SCF-type E3 ubiquitin ligase-proteasome pathway, involving TIR1/AFB receptors, is central to auxin signaling [20,40]. In this study, decreased auxin levels between 7 and 14 DAG coincided with significant upregulation *AFB5* and *AFB2* (Figure 3E,F), genes implicated in SCF-dependent proteolytic metabolism. Elevated AFB expression likely primes the system for rapid auxin signaling despite low IAA levels, facilitating the transition from wound healing to cell proliferation.

Rootstock-specific upregulation of *NDPK2* (Figure 3H)—a modulator of auxin transport—suggests localized reprogramming of auxin flux. By regulating PIN1 phosphorylation (as in *Arabidopsis*), increased *NDPK2* expression may redirect auxin toward the graft interface, creating gradients essential for cambium activation [41,42]. Concurrently, downregulation of *COV1* and *ETA3* (Figure 3D,G)—genes involved in auxin-activated signaling—likely reduces auxin efflux, concentrating residual IAA to promote localized cell division rather than long-distance transport. Together, these dynamics illustrate auxin’s dual role: early depletion facilitates CK-dominant callus proliferation, whereas later rebound (coinciding with *AFB* upregulation) primes vascular differentiation, underscoring the hormone’s pivotal role in orchestrating graft union phases.

### 3.2. Role of CK in Callus Formation After Grafting in Hickory

CK are fundamental to wound union and subsequent differentiation processes [8]. Evidence suggests that initial post-injury stages are characterized by increased concentrations of various CK derivatives, which are integral to the healing process [43,44,45]. In hickory, tZ levels peaked at 7 DAG before declining at 14 DAG, with tZ and its precursor tZR showing maximum accumulation in both rootstocks and scions at 7 DAG. This pattern aligns with observations in oil tea (*Camellia oleifera* Abel.) [34] and *Arabidopsis* [46], where CK facilitate callus initiation and proliferation. Anatomical investigations have revealed that callus formation in hickory occurs mainly between 7 and 14 DAG, coinciding with isolation layer breakdown and increased cell division [22]. The elevated tZ levels during this period aligned with a role for CK in promoting cell division and callus formation, thus enhancing graft survival. Our transcriptomic analysis results revealed enhanced expression of CK-related genes, including the *IPT3* gene for CK synthesis at 7 and 14 DAG [45]. Additionally, the *ABCG14* root-to-shoot CK transporter showed significant upregulation at 14 DAG in rootstocks [46]. The rice homolog *OsABCG18* similarly regulates long-range CK transport [47], suggesting that CK movement from roots to shoots may be important for successful grafting.

*IPT3* upregulation (Figure 5B) directly correlated with elevated CK levels (Figure 1F), confirming its role as the rate-limiting enzyme in biosynthesis. Sustained *IPT3* expression in both tissues likely reflects wound-induced CK activation, consistent with *Arabidopsis* and *tobacco* studies linking *IPT* genes to CK-driven cell proliferation [48,49]. Rootstock-specific *ABCG14* upregulation (Figure 5H) underscores its function in CK transport, generating gradients to direct CK toward the graft interface for localized cell division. Coordinated induction of type-A response regulators (*RR3* and *ARR4*) and *ZFP5* (Figure 5A,F,G) reflects active CK signaling, with type-A RRs forming feedback loops to balance proliferation and differentiation. Tissue-specific *LOG* gene expression (Figure 5C–E) reveals nuanced CK regulation: systemic *LOG5* upregulation supports broad CK activation, whereas *LOG1*/*LOG4* divergence (root-up/scion-down) suggests localized control of CK riboside hydrolysis. Elevated *LOG1*/*LOG4* in rootstocks may enhance CK bioavailability for callus initiation; in comparison, their suppression in scions prevents hyperplasia from excessive CK accumulation. Together, these dynamics highlight CK’s pivotal role in coordinating wound healing and vascular differentiation, thereby prioritizing meristematic growth during critical graft union phases.

### 3.3. Interaction Between ETH and Auxins During Hickory Grafting

The gaseous phytohormone ETH serves a vital role in plant growth, development, and stress responses. Its biosynthesis is activated at wound sites of plant grafts, aiding in the healing and union processes [50,51,52]. In this study, the results of biochemical analyses revealed a progressive increase in ACC concentration, reaching twice the initial level by 14 DAG, which aligns with transcriptomic upregulation of the ETH biosynthesis gene *ACS1* in both rootstocks and scions. *ACS1*, encoding the rate-limiting enzyme in ETH synthesis, converts S-adenosyl-L-methionine to ACC, which is subsequently oxidized to ETH by ACC oxidase. Notably, *ACO1* expression increased in rootstocks, while whereas *ACO5* showed scion-specific upregulation (Figure 8A,C), indicating tissue-specific modulation of ETH production. These findings corroborate grafting studies in tobacco, wherein elevated ACC levels at the wound site promoted isolation layer formation and union establishment [19].

ETH signaling was further activated through the coordinated induction of key regulatory components. The MAPKK family member *MKK9*, which links ETH biosynthesis to stress responses [35], demonstrated substantial upregulation in both tissues. Concurrently, ETH-responsive ERF/AP2 transcription factors *ESE1* and *ESE3* (ERF B-6 subfamily) were induced at 14 DAG (Figure 8E,F), paralleling observations in *Arabidopsis* wherein *ERF1* promotes auxin biosynthesis by activating WEAK ETHYLENE INSENSITIVE2 (*WEI2*) [23]. This regulatory cascade is consistent with the well-established role of ETH acting upstream of auxin signaling. Previous studies have indicated that ETH enhances auxin biosynthesis and root growth regulation in *Arabidopsis* by directly promoting the expression of auxin-related genes [53,54]. In hickory, *ESE* upregulation may similarly regulate auxin-related processes, facilitating vascular bundle differentiation during later graft stages.

### 3.4. Necessity of Low SL Concentrations for Hickory Grafting

SL, carotenoid-derived molecules, are instrumental in the regulation of plant development and architecture [55]. In hickory, a pronounced reduction in SL concentration was observed post-grafting, with levels decreasing by approximately 4.5-fold in rootstocks and 8.4-fold in scions at 7 DAG (Figure 1B). This dramatic decline aligns with transcriptomic and qRT-PCR data showing demonstrating coordinated modulation of SL pathway genes. The α/β hydrolase receptor *D14,* essential for SL perception, was significantly upregulated at both 7 and 14 DAG, while whereas the SL-responsive suppressor gene *SMAX1* exhibited marked downregulation during the same period (Figure 9A–C). These molecular dynamics suggest a negative feedback mechanism in which reduced SL bioavailability triggers compensatory *D14* induction to enhance tissue sensitivity to residual SL, thereby optimizing signaling efficacy.

Research on SL-deficient mutants in *Arabidopsis* has shown reduced formation layer activity, which SL application could restore, indicating the influence of SL on cell division within the formation layer [36]. Despite these findings, the specific role of SL in the wound response remains unclear. Grafting experiments in pea (*Pisum sativum* L.) have highlighted the role of SL in suppressing branch development, serving as secondary messengers for auxin in the maintenance of apical dominance [56]. This interaction suggested a regulatory mechanism by which auxin modulates SL and CK biosynthesis to control stem branching [55].

Of note, our study revealed that SL levels and *SMAX1* expression at hickory graft interfaces progressively declined below pre-grafting (0 DAG) levels during the observation period. These findings imply that the proliferation of hickory calluses and the differentiation of vascular bundles require low concentrations of SL. The successful use of SL-deficient mutants in grafting suggests that SL may exert a negative regulatory effect on graft union processes [57,58], highlighting the complex interplay of hormonal interactions necessary for successful grafting.

## 4. Materials and Methods

### 4.1. Plant Materials and Growth Conditions

The experiment was conducted in a controlled environment greenhouse maintained at 25 ± 3 °C, with relative humidity of 60–70%, a 12 h light/12 h dark photoperiod, and a photosynthetic photon flux density of 500–700 µmol·m^−2^·s^−1^. Two-year-old hickory plants (var. ‘Zhelinshan 1’, Figure S1) of uniform size (height: 40–50 cm; stem diameter: 8–10 mm) were selected as rootstocks and cultivated in pots containing 10 kg of soil mixture (3:1 peat:perlite (*v*/*v*) with 2 kg/m^3^ controlled-release fertilizer (STANLEY^®^, Shanghai, China)). The seedlings were irrigated every five days.

### 4.2. Hickory Grafting and Sample Collection

Grafting was performed in April using two-year-old plants as rootstocks and 7–8 cm annual branches with buds from a 15-year-old hickory tree (var. ‘Zhelinshan 1’) as scions, ensuring genetic uniformity within the clone population. Skilled technicians pruned the rootstocks to a length of 8–10 cm and grafted them with annual branch scions, wrapping the joints in polyethylene film to promote successful union (see Figure S2). For each time point (0, 7, and 14 DAG), five grafts were randomly selected for sampling after grafting. Samples were collected from the graft union interface, encompassing slices from both the rootstocks and scions, which were excised using a sharp, sterile single-sided blade to a thickness of approximately 1 mm. Samples from rootstocks and scions were collected separately. Immediately following collection, the samples were immersed in liquid nitrogen and stored at −80 °C for subsequent analyses.

### 4.3. Determination of Hormone Concentrations

#### 4.3.1. Sample Preparation and Extraction

For extraction, 50 mg of ground sample was mixed with 1 mL of a solvent mixture (methanol (Sigma-Aldrich^®^, St. Louis, MO, USA), water, and formic acid (Sigma-Aldrich^®^, St. Louis, MO, USA); 15:4:1) and 10 μL of an internal standard (100 ng·mL^−1^) (Table S3). The mixture was centrifuged at 12,000 rpm for 5 min at 4 °C to separate the soluble fraction. After centrifugation, the supernatant was evaporated to remove the solvent, reconstituted in 80% methanol, and filtered through a 0.22 μm membrane for LC–MS/MS analysis [59,60].

#### 4.3.2. Ultra-Performance Liquid Chromatography (UPLC)

During our analysis, we employed a UPLC–Electrospray ionization–tandem mass spectrometry (ESI–MS/MS) system (QTRAP 6500+, SCIEX, Framingham, MA, USA) with a Waters ACQUITY UPLC HSS T3 C18 column (Waters, Milford, MA, USA). The solvent system included water with 0.04% acetic acid (solvent A) and acetonitrile with 0.04% acetic acid (solvent B). The gradient program began with 5% solvent B for the first minute, increased linearly to 95% solvent B from 1 to 8 min, maintained at 95% solvent B for 8 to 9 min, and then returned to 5% solvent B for a total of 12 min. The analysis involved the use of a 2 μL injection volume, as supported by evidence from a previous work [61,62,63].

#### 4.3.3. ESI–MS/MS Conditions

Phytohormones were detected using a QTRAP 6500+ LC–MS/MS system in positive and negative ionization modes. Parameters included a source temperature of 550 °C and ion spray voltages of 5500 V for positive mode and −4500 V for negative mode. Phytohormones were identified using a customized multiple reaction monitoring (MRM) program. Data were collected using Analyst software version 1.6.3, and metabolites were analyzed with Multiquant software version 3.0.3, enabling precise measurement of phytohormone levels in the samples [64,65,66].

### 4.4. RNA-Sequencing

For sequencing, total RNA was extracted from the sample using an mRNA Mini Kit (Qiagen, Hilden, Germany). RNA was digested using Turbo-DNase (Ambion, Foster City, CA, USA). RNA quality was evaluated on a NanoPhotometer^®^ spectrophotometer (IMPLEN, München, Germany). The integrity and quantity of total RNA were assessed using a BioAnalyzer (Agilent Technologies, La Jolla, CA, USA). RNA-Seq was performed by LC-bio (Hangzhou, China) on the Illumina HiSeq 4000 platform. Raw reads were trimmed with Trimmomatic (v0.39; ILLUMINACLIP:2:30:10, LEADING:20, TRAILING:20, SLIDINGWINDOW:4:15). Q20, Q30 and GC contents of the clean data were calculated. For alignment to the hickory reference genome (http://www.juglandaceae.net/ (accessed on 5 March 2024)), we used HISAT2 (v2.2.1; --dta --rna-strandness RF) with E-value < 1 × 10^−5^. Differential expression analysis involved the use of DESeq2 (v1.36.0; adjusted *p*-value < 0.05, |log2FC| > 1). Functional annotation of genes was performed using two public databases: KEGG (The Kyoto Encyclopedia of Genes and Genomes) and GO (Gene Ontology). Gene expression level was evaluated based on fragments per kilobase of exon model per million mapped reads (FPKM) (FDR < 0.05). The heat map representing the transcriptomic analysis was created by using TBtools (v2.310) to gain insights into gene expression dynamics in these pathways [67].

### 4.5. qRT-PCR Analysis

Total RNA was extracted from rootstock and scion tissues using a MiniBEST Plant RNA Extraction Kit (Takara, Dalian, China). Reverse transcription reactions were performed using the PrimeScriptTM RT Reagent Kit (Takara, Dalian, China) following the manufacturer’s protocol. Target gene expression levels were quantified via qRT-PCR with TB Green^®^ Premix Ex Taq ™ II FAST qPCR (Takara, Dalian, China). Primer sequences for qRT-PCR analysis were designed using Primer Premier 5.0 software, with β -actin serving as the internal control (Table S2). The thermal cycling parameters were as follows: 95 °C for 30 s, 40 cycles at 95 °C for 5 s, and 60 °C for 10 s. All assays included three independent biological replicates, each with three technical replicates. Relative gene expression levels were calculated using the 2 ^−∆∆Ct^ method.

### 4.6. Statistical Analysis

Statistical analyses were performed using IBM SPSS Statistics 26.0, and graphs were created with GraphPad Prism 8.0. Significant differences were identified though analysis of variance (ANOVA) with a significance threshold of *p* < 0.05. Data are shown as the mean ± standard error (SE).

## 5. Conclusions

In summary, in this study, we have elucidated the dynamic hormonal interactions occurring during the early stages of hickory graft union formation. The hormones auxin, CK, ETH, JA, and SLs exhibit spatially and temporally regulated changes that orchestrate defense responses and tissue regeneration. An early reduction in auxin levels is associated with altered expression of biosynthesis genes (*TAR2* and *MES17*), indicating a transition from vascular development to wound-induced cell proliferation. The accumulation of CK, driven by the activation of *IPT3* and *LOG*, corresponds with meristem activation and callus growth. The biosynthesis (*ACS1* and *ACO*) and signaling (*MKK9*) pathways of ETH facilitate stress adaptation and tissue fusion. Systemic suppression of JA (via downregulation of *AOC4* and *AOS*) and alterations in SL biosynthesis (*D14*) and SL signaling (*SMAX1*) reflect a trade-off between reduced defense and enhanced regenerative growth. These hormonal interactions promote vascular reconnection, as evidenced by increases in tZ and ACC levels and the induction of *ABCG14*. Tissue-specific responses, such as the reduction in JA in the scion and the decline of SL in the rootstocks, underscore the complex communication occurring at the graft interface. These findings advance the understanding of hormone-mediated mechanisms underlying the early stages of graft union formation.

## Data Availability

Data will be made available on request.

## Supplementary Materials

The following supporting information can be downloaded at: https://www.mdpi.com/article/10.3390/plants14142229/s1. Table S1. Differentially expressed genes involved in hormone biosynthesis and signaling pathways in hickory rootstocks and scions at 0, 7, 14 DAG with corresponding FPKM Values. Table S2. Primers used in this study. Table S3. Chemical information for internal standard solution preparation. Figure S1. Phenotype of hickory ‘Zhelinshan 1’. Figure S2. Schematic diagram of hickory grafting procedure.

## Abbreviations

The following abbreviations are used in this manuscript:

CK	cytokinins
ETH	ethylene
ABA	abscisic acid
JA	jasmonic acid
ACC	1-aminocyclopropane-1-carboxylate
ARR	Arabidopsis response regulator
AVG	aminoethoxyvinylglycine
GA	gibberellin
*ARF*	auxin response factor
DAG	days after grafting
tZ	trans-Zeatin riboside
IAA-Asp	IAA-aspartate
IAA-Ala	IAA-alanine
IAN	Indole-3-acetonitrile
ICA	Indole-3-carboxylic acid
ICAld	indole-3-carbaldehyde
TRP	tryptophan
IPA	Indole-3-pyruvic acid
MEIAA	Methoxy-indole-3-acetic acid
*TAR2*	tryptophan aminotransferase related 2
*MES17*	methylesterase 17
*NDPK2*	nucleoside diphosphate kinase 2
cZ	cis-zeatin
cZR	cis-zeatin riboside
DZ	cZ-riboside dihydrozeatin
DZHR	dihydrozeatin ribonucleoside
DHZ7G	dihydrozeatin-7-glucoside
DHZROG	dihydrozeatin-O-glucoside riboside
IPR	N6-isopentenyladenosine
tZOG	trans-zeatin-O-glucoside
tZR	trans-zeatin riboside
mT9G	meta-Topolin-9-glucoside
OPDA	Oxophytodienoic acid
MEJA	methyl jasmonate
*AOS*	oxide synthase
*AOC4*	allene oxide cyclase 4
ACO	ACC oxidase
UPLC	Ultra-performance liquid chromatography
ESI–MS/MS	Electrospray ionization–tandem mass spectrometry
