# Supplementary material for: Hormone Fluctuation and Gene Expression During Early Stages of the Hickory Grafting Process
Source: Plants (Basel). 2025 Jul 18;14(14):2229. doi: 10.3390/plants14142229 (PMC12298595; doi:10.3390/plants14142229)
Supplement: Supplementary file 1 [file plants-14-02229-s001.zip › plants-3655817-supplementary/plants-3655817-supplementary/Supplementary Figures.pdf]

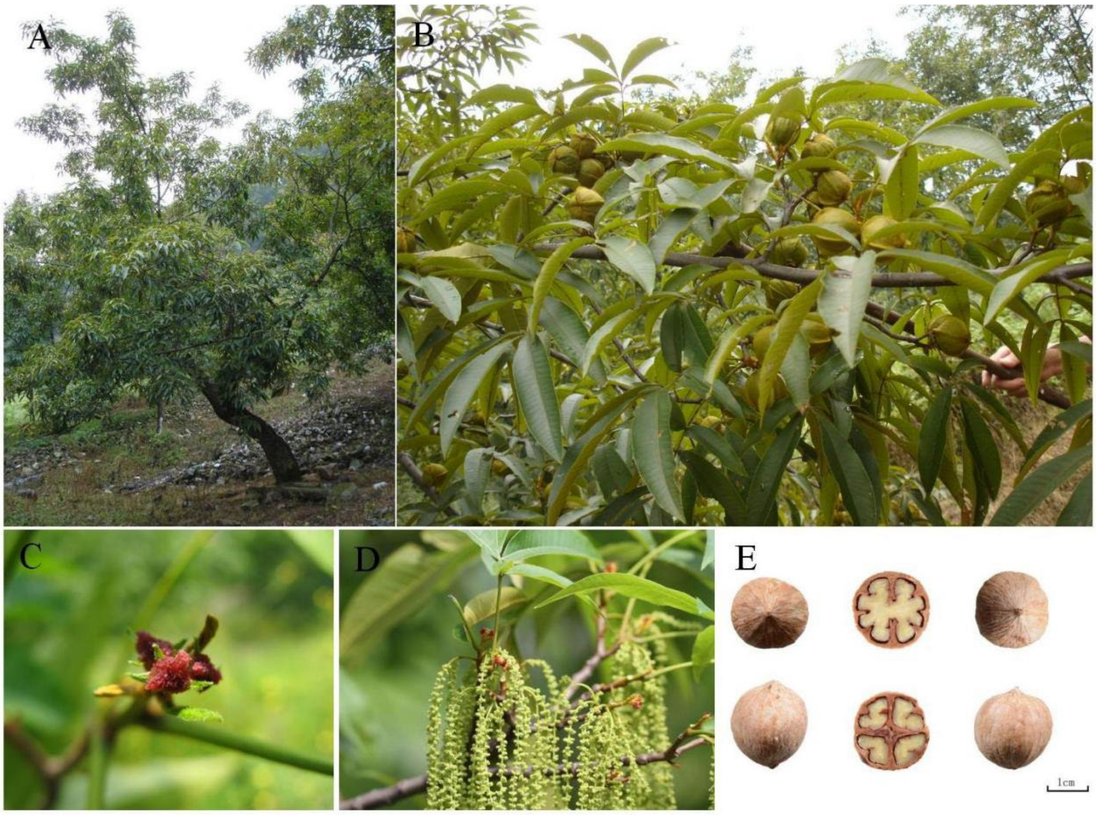

Figure S1. Phenotype of hickory‘Zhelinshan 1’. (A) Mature tree of hickory ‘Zhelinshan 1’ in its native habitat (Tianmu Mountain, Zhejiang Province, China). (B) Fruiting traits of ‘Zhelinshan 1’. (C) Female flowers of ‘Zhelinshan 1’. (D) Male catkins (ament) with numerous stamens of ‘Zhelinshan 1’. (E) Nut morphology of ‘Zhelinshan 1’.

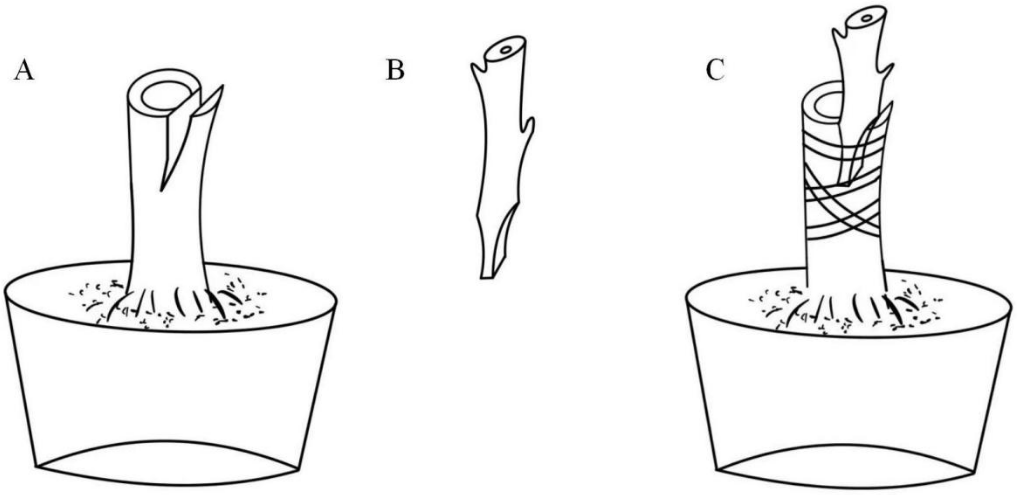

Figure S2. Schematic diagram of hickory grafting procedure. (A) Rootstock preparation. (B) Scion preparation. (C) Graft union formation in hickory.
